# Stable 3D Deep Convolutional Autoencoder Method for Ultrasonic Testing of Defects in Polymer Composites

**DOI:** 10.3390/polym16111561

**Published:** 2024-05-31

**Authors:** Yi Liu, Qing Yu, Kaixin Liu, Ningtao Zhu, Yuan Yao

**Affiliations:** 1 Institute of Process Equipment and Control Engineering, Zhejiang University of Technology, Hangzhou 310023, China; yliuzju@zjut.edu.cn (Y.L.); 2112102164@zjut.edu.cn (Q.Y.); 2 The State Key Laboratory for Electronic Testing Technology, North University of China, Taiyuan 030051, China; 3 Xi’an Zhanshi Testing & Engineering Co., Ltd., Xi’an 710000, China; zhuningtao270@163.com; 4 Department of Chemical Engineering, National Tsing Hua University, Hsinchu 300044, Taiwan

**Keywords:** carbon-fiber-reinforced polymer, ultrasonic testing, 3D convolution, deep autoencoder, receptive field

## Abstract

Ultrasonic testing is widely used for defect detection in polymer composites owing to advantages such as fast processing speed, simple operation, high reliability, and real-time monitoring. However, defect information in ultrasound images is not easily detectable because of the influence of ultrasound echoes and noise. In this study, a stable three-dimensional deep convolutional autoencoder (3D-DCA) was developed to identify defects in polymer composites. Through 3D convolutional operations, it can synchronously learn the spatiotemporal properties of the data volume. Subsequently, the depth receptive field (RF) of the hidden layer in the autoencoder maps the defect information to the original depth location, thereby mitigating the effects of the defect surface and bottom echoes. In addition, a dual-layer encoder was designed to improve the hidden layer visualization results. Consequently, the size, shape, and depth of the defects can be accurately determined. The feasibility of the method was demonstrated through its application to defect detection in carbon-fiber-reinforced polymers.

## 1. Introduction

Carbon-fiber-reinforced polymers (CFRPs) [1] combine the excellent properties of both carbon and fiber materials and are widely used in aerospace, new energy, military applications, and other fields [2]. However, defects may arise during the manufacture and use of these materials. These defects not only affect the comprehensive characteristics of the material and reduce its load-bearing capacity but also have a significant impact on product quality [3]. Therefore, regular nondestructive testing (NDT) of CFRP is crucial to ensure the structural quality of the material. Currently, the mainstream NDT methods include ultrasonic testing (UT) [4], eddy current testing [5], and infrared thermography [6]. UT is widely used because of its nonpolluting and noninvasive nature, ease of operation, accurate positioning, and high reliability [7,8].

However, ultrasound signals are affected by surface echoes, bottom echoes, and noise, which interfere with the judgment of defects, making them difficult to identify [9]. Recently, several methods have been proposed for analyzing ultrasonic inspection data to improve their effectiveness. One-dimensional (1D) signal filtering processing methods, including empirical wavelet transform [10], short-time Fourier transform analysis [11], and Wigner–Ville distribution [12], are mainly used for the analysis of ultrasound signals. These methods can extract the characteristic information of defects but lack visualization. Image defect detection methods include support vector machines [13], manifold learning algorithms [14], and feature-selective clustering [15]. These machine learning methods can improve the identifiability of defects. For ultrasonic data with considerable noise and large echoes, deep learning [16,17] methods are more attractive. Models such as neural networks can automatically extract features and therefore contribute to the development of defect detection.

Recently, the rapid development of deep learning has led to its successful application in several manufacturing and maintenance industries [18]. An autoencoder (AE), a typical unsupervised deep learning model, has shown good performance in extracting potential features from high-dimensional data and eliminating redundant information. Some AE variants have made significant progress in NDT data analysis. Convolutional AE (CAE) [19] can use activation functions to extract nonlinear features, which helps analyze nonlinear, multinoise ultrasonic data [20]. The deep AE (DAE) [21] was successfully employed to analyze infrared thermography defect signals, and the output of the intermediate hidden layer was visualized to accentuate the defects. However, it focuses more on analyzing images and pixel data and often ignores important time-series information. Three-dimensional convolution (3D-conv) [22] can accurately extract deep spatial features from images, significantly outperforming previous feature extraction models based on 1D and two-dimensional (2D) information. However, few studies have applied 3D-conv for defect detection in CFRP.

In this study, a stable 3D deep convolutional autoencoder (3D-DCA) was developed to improve the accuracy and reliability of defect detection. To address the issue of significant ultrasonic noise, a 3D convolutional neural network (CNN) is used to simultaneously extract the spatiotemporal features of the data and reduce noise in the ultrasonic data. The depth-wise receptive field in the hidden layers of the autoencoder maps the defect information to its original depth position, thereby reducing the impact of surface and bottom reflections. Additionally, a two-layer encoder was developed to enhance the visual representation of the hidden layers. The intersection over union (IoU) [23] and contrast-to-noise ratio (CNR) [24] were used to evaluate the effectiveness of the proposed approach.

The remainder of this paper is organized as follows. Section 2 presents the ultrasonic data structure and preprocessing. Section 3 describes the stable 3D-DCA model framework and its implementation. In Section 4, a CFRP specimen is presented and the experimental results are discussed. Finally, the conclusions are presented in Section 5.

## 2. Data Collection and Preprocessing

### 2.1. Ultrasound Scanning

**Figure 1:**
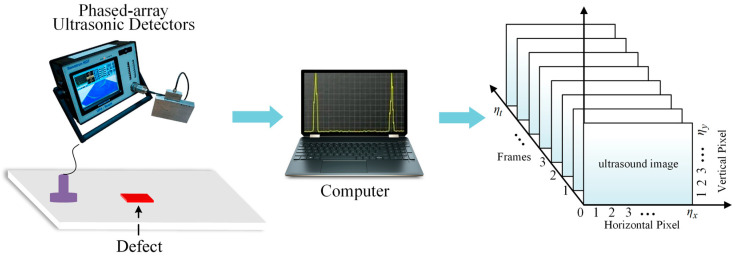
Schematic of a phased-array ultrasonic acquisition system.

Phased-array ultrasonic detectors utilize multichip probes, where each probe functions as a separate transmitter or receiver array. The process operates as shown in Figure 1. The transmitter array directs the emission of the ultrasound signals from each unit to form the desired acoustic beam [25]. Simultaneously, the receiver array manages the timing of each unit to capture reflected signals. The ultrasound waves propagate through the test object and are reflected off the internal defects or interfaces. The strength of the reflected signals correlates with the properties of the materials on either side of the interface [26]. Thus, the ultrasound echoes that return from the workpiece can be used to locate defects within the material.

The collected ultrasonic data were then converted into a 3D matrix for processing with a stable 3D-DCA. The ultrasonic time series served as the depth dimension, with the horizontal and vertical pixels representing the height and width dimensions of the ultrasound image, respectively. Consequently, the convolution operation extracts feature information from the time series of multiple ultrasound images, thereby reducing the reliance on manual screening operations.

### 2.2. Data Structures and Preprocessing

The dataset acquired during ultrasonic inspection typically comprises a 3D matrix that displays planar projections of ultrasound echoes at various depths of the specimen. This is achieved by setting a specific depth range to acquire an *n_h_* frame echo signal. Each image frame consists of *n_x_* × *n_y_* pixels. The 3D ultrasonic matrix $X\in R^{T\times M\times N}$ is then transformed into a 2D matrix $X_{1}\in R^{T\times R}$, where *M*, *N*, and *T* denote the length, width, and number of frames, respectively, and *R = M* × *N*.

After converting the 3D ultrasonic matrix into a 2D matrix, it was observed that the amplitude of the echoes at shallow defects was usually larger than that at deep defects, and the amplitudes of the surface and bottom echoes exceeded those of the defect echoes [27]. To mitigate the effects of differences in scale, it is often necessary to preprocess the original ultrasonic data x*_i,j_*, where x*_i,j_* is the original value at the *j*th pixel in column *i* of **X**_1_. In this study, the robust normalization [28] method was applied, which is calculated as follows:

(1) $$x_{i,j}^{\prime }=\frac{x_{i,j}-med}{IQR},$$

where $x_{i,j}^{\prime }$ represents normalized data. *med* is the median of x*_i_*, and *IQR* is the interquartile range of x*_i_*. The effects of outliers in the data can be reduced using robust normalization techniques.

## 3. Methodologies

### 3.1. Description of 3D-DCA

An AE typically consists of encoder and decoder networks. The encoder compresses the data dimensions to extract potential features, and the decoder reconstructs the input data based on these features. As previously mentioned, $X\in R^{T\times M\times N}$ represents a set of ultrasonic data, and the feature extraction process using 3D-DCA is represented as

(2) $$\begin{array}{l} X_{L}=f(W_{\mathrm{en}}⊙X+b_{\mathrm{en}})\\ \hat{X}=f(W_{\mathrm{de}}⊙X_{L}+b_{\mathrm{de}}) \end{array},$$

where $X_{L}\in R^{T\times M\times N}$ (*t* << *T*) denotes the output of the encoder, *t* is the number of feature maps, ⊙ represents a 3D-conv operation, **W**_en_ and **b**_en_ represent the encoder weight and bias, respectively, $\hat{X}\in R^{t\times M\times N}$ denotes the ultrasonic data reconstructed by the decoder, and **W**_de_ and **b**_de_ represent the decoder weight and bias.

Compared with 2D convolution, the use of 3D-conv in this model accounts for temporal information. The dimensions of the 2D convolution can be expressed as *k_h_* × *k_w_*, whereas those of 3D-conv are *k_h_* × *k_w_* × *k_d_*. The formula for 3D-conv is similar to that for 2D convolution, with the primary distinction being that the convolution operation extends across three dimensions—depth, width, and height—that require manipulation in a volumetric space. Thus, 3D-conv can extract both spatial and temporal information, thereby enhancing defect detection accuracy. Similar to the 2D convolutional network structure, 3D-DCA includes an encoder, decoder, and loss function, but with the 2D convolutional kernel replaced by a 3D-conv kernel. Assuming that the feature map of layer *l*-1 is A*_l_*_-1_, the representation of the value of the *i*th feature of layer *l* at position (*x*, *y*, z) is

(3) $$A_{l,i}^{xyz}=f\left(\sum_{c=0}^{C_{L}-1}\sum_{h=0}^{H_{L}-1}\sum_{w=0}^{W_{L}-1}\sum_{d=0}^{D_{L}-1}W_{l,i}^{chwd}A_{l-1}^{c(x+h)(y+w)(z+d)}+b_{l,i}\right),$$

where $W_{l,i}^{chwd}$ denotes the value of the *i*th convolution kernel connected to layer *l* at a position (*h*, *w*, and *d*); *c* denotes the feature map fed from layer *l*-1 to layer *l*; *H*, *W*, and *D* denote the height and width of the 3D-conv kernel and the depth in the time series, respectively; $b_{l,i}$ denotes the bias of the *i*th feature map in the *l*th layer; *f*( ) denotes the activation function; and the activation function is Leaky Relu (LReLu).

### 3.2. Stable 3D-DCA for Ultrasonic Defect Detection

In this section, the construction steps of the stable 3D-DCA model to improve the visibility of defects are described in detail. A phase-controlled ultrasonic detector was used to acquire ultrasonic data, and a stable 3D-DCA method was applied to extract defective regions from the image. The main steps of the stable 3D-DCA model include 3D-conv for ultrasonic signal denoising, RF-based defect depth prediction, and enhancement of detection performance.

#### 3.2.1. 3D-Conv for Ultrasonic Denoising

The ultrasound images acquired using the phase-controlled ultrasonic detector exhibited significant redundancy. The unfolded data matrix **X_1_** is preprocessed using the robust normalization method described in Section 2.2 to improve the data presentation. After preprocessing, the 2D data **X_1_** were converted into a 3D matrix $X\in R^{T\times M\times N}$.

**Figure 2:**
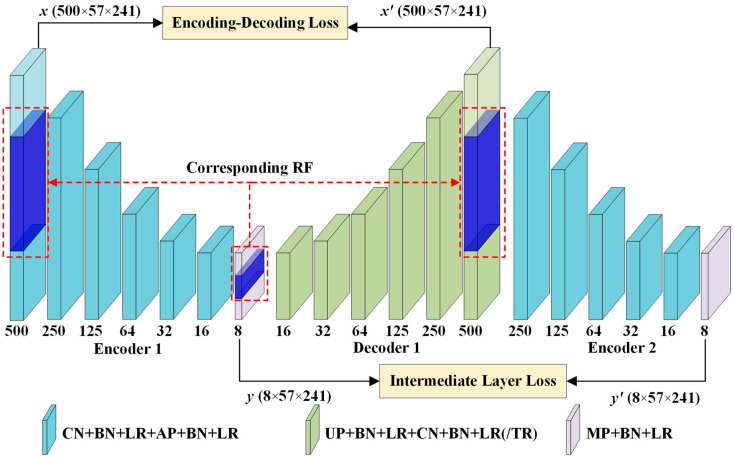
Stable 3D-DCA flowchart. CN represents 3D-conv, BN represents batch normalization, LR represents Leaky ReLU, TH represents Tanh used in the output layer of the decoder, AP represents 3D average pooling, UP represents upsampling, and MP represents 3D maximum pooling.

The structure of a stable 3D-DCA model is shown in Figure 2. The stable 3D-DCA core architecture comprises two encoders and a decoder.

The encoder has six coded convolutional blocks, each consisting of a convolutional layer and a pooling layer. The 3D filter can move in all three directions of the ultrasonic data and detect features both vertically and horizontally. At each position, a convolved value was calculated to attenuate noise in the original ultrasonic data and clarify the spatial information of the defects. To obtain an enhanced image with low noise, the convolution kernel of the convolutional layer was set to 3 × 3 × 3 and the step size was set to 1 × 1 × 1. Then, 3D maximum pooling was used in the last layer of the encoder, whereas 3D average pooling was used in the other layers. The kernel of the pooling layer was 3 × 3 × 3. Using 3D-conv, the ultrasonic time series can be processed directly in the ultrasound processing tasks without segmenting the 3D data into multiple ultrasound images. Additionally, to accelerate the mapping of the RF and reduce the number of parameters used in the model, the step size of the pooling layer was set to 2 × 1 × 1, focusing only on feature extraction in the depth direction. The Adam optimizer and dropout strategies were used to prevent model overfitting and reduce computation.

The decoder incorporates an upsampling layer to resize the reconstructed map and obtain a noise-reduced map that corresponds one-to-one to the input data. The decoding layer included six convolutional blocks, each featuring an upsampling layer and a convolutional layer. A batch normalization layer and an activation function were added to each layer of neurons, with the tanh activation function [29] used for the output layer of the decoder and LReLU for the other layers.

The ultrasonic data possess a 3D structure. The stable 3D-DCA denoising effect is highlighted by the integration of components such as 3D-conv and 3D pooling.

#### 3.2.2. RF-Based Defect Depth Prediction

**Figure 3:**
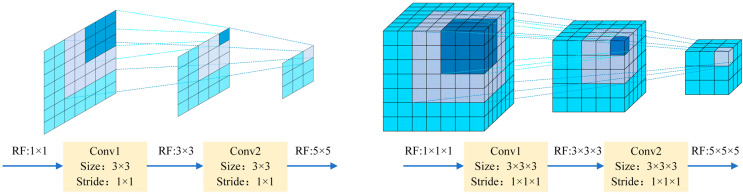
RF mapping of 2D convolution vs. 3D-conv.

In a 3D-conv neural network, the RF refers to the volume of input data that a point on the feature map can perceive. As illustrated in Figure 3, the larger the RF value of the neuron, the wider the range of the original data it can access. The RF of the current layer is calculated as follows:

(4) $$RF_{i+1}=RF_{i}+(k-1)\times S_{i},$$

where *RF_i+_*_1_ denotes the RF of the current layer; *RF_i_* denotes the RF of the previous layer; *k* denotes the size of the convolution kernel; and *S_i_* represents the product of the step sizes of all previous layers (excluding the current layer). The formula used is as follows:

(5) $$S_{i}=\prod_{i=1}^{i}Stride_{i},$$

Using the data from this study as an example, the network framework and RF calculations are presented in Table 1. The RF of each point on the feature map can be determined using Equation (4): the ultrasonic surface and bottom echoes could be bypassed by adjusting the RF size. Consequently, RF can be utilized to approximate the CFRP defect information for each layer and mitigate the impact of surface and bottom echoes on the results.

#### 3.2.3. Improving the Detection Results

Feature extraction using an encoder compresses numerous ultrasound images into a low-dimensional space, thereby facilitating easier defect detection. However, without stringent parameter constraints, the effectiveness of the extracted features cannot be ensured. In this study, the outputs of the two encoders are used to construct the intermediate layer loss. This adjustment ensured that the defect size and shape closely resembled the ground truth.

(6) $$\mathrm{Loss}=\mathrm{MSELoss}\left(x,x^{\prime }\right)+\mathrm{MSELoss}\left(y,y^{\prime }\right)$$

Defect recognition is a target detection task that focuses on the location, shape, and number of defects. The raw 3D data encoded by stable 3D-DCA resulted in a new 3D matrix, which was decomposed into several 2D matrices of size *x* × *y*. These can be visualized as 2D images for enhanced defect visualization.

A comparison between the stable 3D-DCA and DAE highlighted several advantages. First, a stable 3D-DCA learns the spatial and temporal features of ultrasonic data, whereas the DAE overlooks the depth and potentially original image information. Second, stable 3D-DCA approximates the depth order of the defect information through the mapping between the deep feature, RF, and original image, making defect detection more meaningful. In addition, the loss function designed for a stable 3D-DCA ensures better results.

## 4. Experimental Results and Discussion

### 4.1. Specimen and Experiment

**Figure 4:**
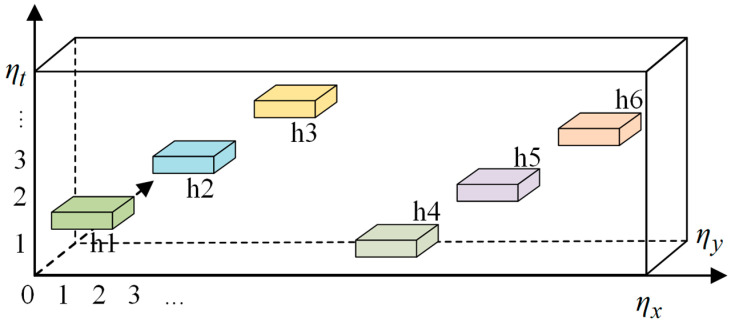
Schematic of the CFRP specimen.

In this study, the ultrasonic detection frequency was 5 MHz and a CFRP sample with artificially preset defects was used to demonstrate the effectiveness of the proposed method. The CFRP was produced by vacuum-assisted resin infusion molding. The carbon fiber material was first cut to dimensions of 241.0 mm × 44.5 mm × 20 mm, and the thickness of each layer was 0.26 mm. Subsequently, six Teflon tapes were embedded within the CFRP at designated locations. As illustrated in Figure 4, each Teflon area measured 400 mm^2^ with a thickness of 0.5 mm. From left to right, defects h2, h3, and h6 were categorized as shallow, whereas h1, h4, and h5 were considered deep defects. Thus, defects of varying locations and depths were introduced and became invisible after the epoxy resin injection. The details of the defects are listed in Table 2.

**Figure 5:**
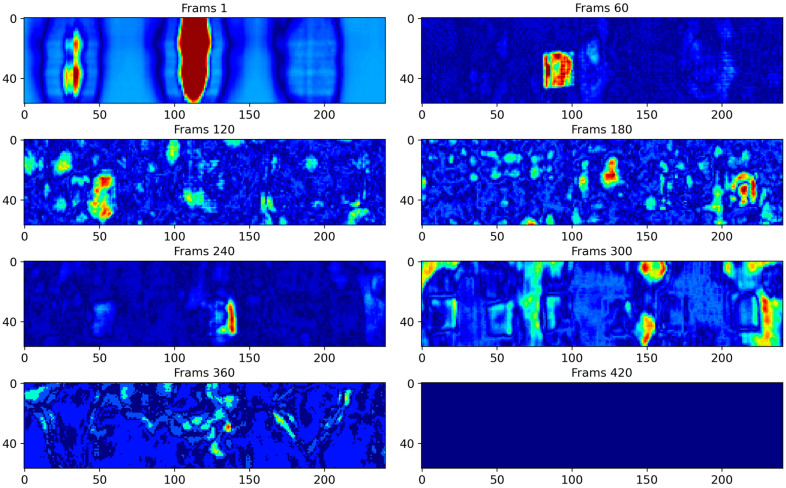
Raw ultrasound images at frames 1, 60, 120, 180, 240, 300, 360, 420.

After the specimen preparation, data were collected using the pulsed-echo method [30] to capture the ultrasound echoes reflected within the workpiece. If defects are present, the ultrasound echo signals will include not only the bottom and surface echoes but also defect echoes in the middle of their range. Experimentally, 500 ultrasonic images were acquired in which severe background noise was observed, complicating defect detection. Each ultrasound image measured 57 × 241, totaling 13,737 pixels. Figure 5 shows the ultrasound echo diagrams at different locations in the CFRP, where the surface and bottom echoes exhibited relatively high background noise levels. Therefore, raw ultrasonic inspection results are generally unsuitable for direct use, underscoring the need to develop effective ultrasonic defect analysis methods.

### 4.2. Evaluation Metrics

To assess the effectiveness of the stable 3D-DCA for defect location extraction, the IoU, which is commonly used in target detection, was employed. The IoU is the overlapping rate of the predicted frame with the original frame, that is, the ratio of their intersection to their union. This is calculated as follows:

(7) $$\mathrm{IoU}=\frac{A\cap B}{A\cup B},$$

where *A* represents the predicted frame and *B* is the original frame. The formula is used to determine the ratio of the intersecting part of the candidate frame to the labeled frame to measure the correlation between the original frame and the prediction. A larger IoU indicates a closer match between the model predictions and the actual situation.

To objectively evaluate the performance of stable 3D-DCA, CNR, which is commonly used in the image field, was adopted as an assessment index. The formula used is as follows:

(8) $$\mathrm{CNR}=\frac{\left|M_{def}-M_{in}\right|}{\sigma_{in}},$$

where *M_def_* is the average pixel value in the defective region, *M_in_* is the average pixel value in the nondefective region, and $\sigma_{in}$ is the standard deviation of the pixels in the nondefective region. The CNR reflects the contrast between the defective and nondefective regions, with a larger CNR indicating a more discernible defect, particularly when the standard deviation of the nondefective region is small.

To evaluate the performance of stable 3D-DCA in ultrasonic data analysis, the IoU focuses on the accuracy assessment of the defect location, and the CNR focuses on the degree of contrast. A comparison of the experimental results using both IoU and CNR makes the evaluation more reliable.

### 4.3. Results and Analysis

To validate the effectiveness of the stable 3D-DCA method, principal component analysis (PCA) [31] and DAE [21] were applied to the same dataset.

**Figure 6:**
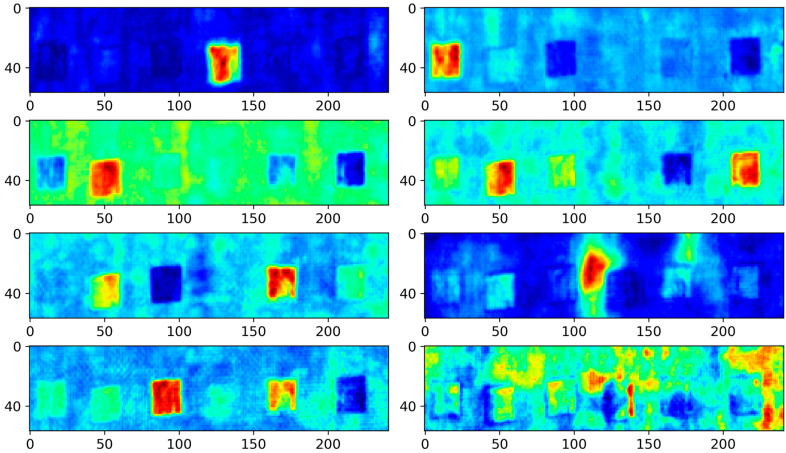
PCA results.

The PCA transforms raw ultrasonic data into a new coordinate system via a linear transformation to optimize the maximum variance in the data. This dimensionality reduction process aims to concentrate most of the defect information within the principal components visually represented in the loading images. However, this concentration inevitably leads to the loss of defect information. Figure 6 shows the loading images produced by the PCA, with colors indicating the values within the loading vector. Although PCA enhances defect visibility compared to the original ultrasonic images, it falls short in clearly delineating the boundaries of each defect. This lack of definition makes the accurate shape recognition of defects challenging. Moreover, PCA-loading images are marred by considerable background noise, further complicating the task of distinguishing defects from their surrounding environment.

**Figure 7:**
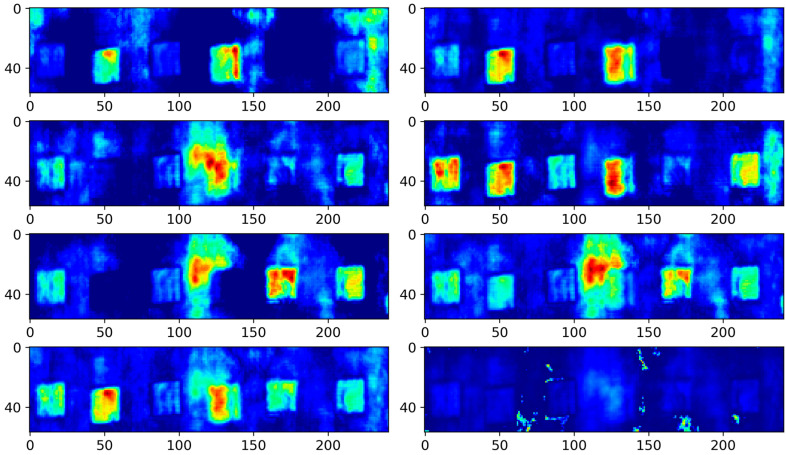
DAE results.

As demonstrated in Figure 7, employing the DAE for feature extraction from ultrasound images and visualizing the encoder’s hidden layer features reveal its effectiveness in infrared thermography applications, but not for noisy ultrasonic data. Although the general shape of the defects was discernible, the overwhelming background noise masked the defect information, with some defect shapes partially obscured.

**Figure 8:**
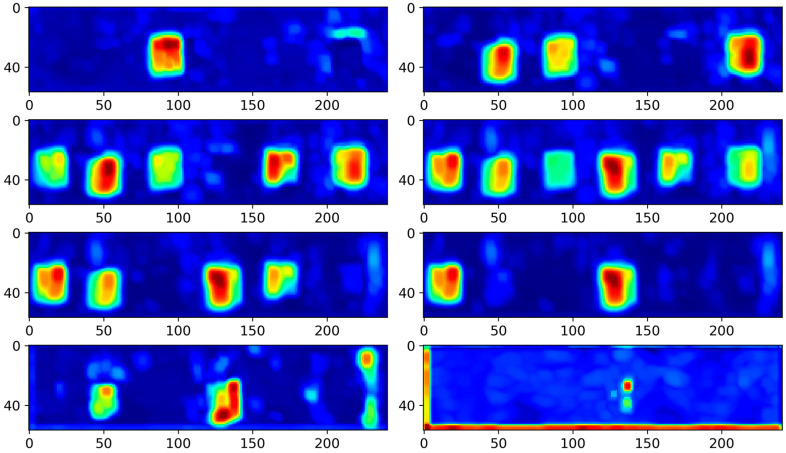
The 3D-DCA results.

Comparative experiments also assessed the performance of 3D-DCA, which utilizes a single-layer encoder and a conventional autoencoder loss function. The number of iterations, batch size, and learning rate were set to 50, 1, and 0.0005, respectively. The encoder performed feature extraction along the depth dimension by compressing 500 images into eight feature maps, each with an RF size of 213 × 27 × 27. This implied that each pixel on the feature map was derived from the aggregation of 213 × 27 × 27 pixel points from the original data, as processed by the model. Therefore, each feature map represented a distinct sensory region within the raw ultrasonic data. The feature maps generated by 3D-DCA bypass the surface and bottom echo characteristics of ultrasound imaging, making the depth order of the defects readily discernible. For instance, in Figure 8, the first feature map highlights the shallowest defect and the second map reveals the three shallowest defects. Moreover, 3D average pooling was utilized to average out the local area features, which smoothed out the ultrasonic surface and bottom echoes post-boundary filling. Conversely, 3D maximum pooling was employed to retain a greater amount of textural information, thereby enhancing the details captured in the feature maps. Figure 8 shows that while defects are more distinctly extracted and the depth order is apparent, the background noise remains noticeable. This results in poorly defined boundaries between the background and the defects.

**Figure 9:**
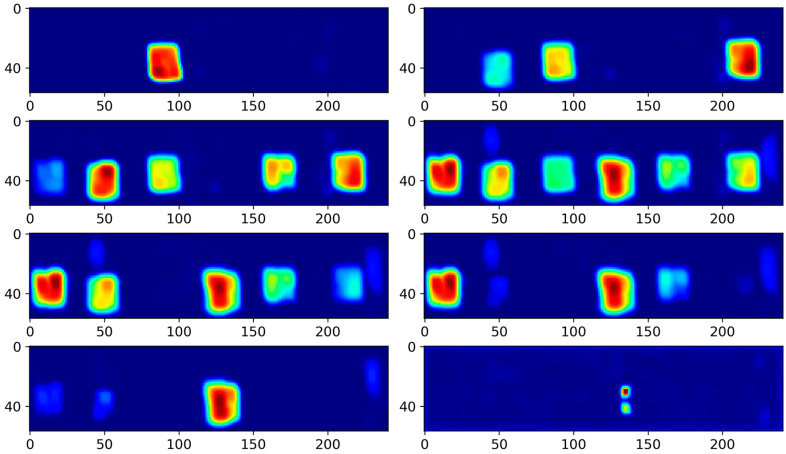
Stable 3D-DCA results.

The 3D-DCA technique highlights that feature extraction using a single-layer encoder may not be sufficient to achieve optimal performance. As illustrated in Figure 2, the enhanced approach, termed stable 3D-DCA, employs a dual-layer encoder to introduce intermediate layer loss. This design encourages the model to reconstruct inputs more accurately, compelling the encoder to learn a more discriminative representation. In Figure 9, the background within each feature map is rendered more smoothly and the defect information stands out more sharply. Compared with the original 3D-DCA model, the stable 3D-DCA demonstrated superior depth-order prediction of defects. For instance, the first feature map in Figure 9 highlights the shallowest defect h3. In the subsequent feature map, h3 becomes less noticeable, h6 gains prominence, and h2 appears but remains faint. This pattern suggests that the initial sequence of defects was h3, followed by h6 and h2. Extrapolating further, the sequence continued with defects h5, h1, and h4. This sequencing can also be deduced in reverse from subsequent feature maps, with the depth-order outcomes presented in Table 3.

To compare the defect extraction capabilities of the different methods, Table 4 presents the IoU values for PCA, DAE, 3D-DCA, and stable 3D-DCA across different defect locations. According to Table 4, the IoU values of stable 3D-DCA were notably superior to those of DAE and PCA. This result is consistent with the visualization results, demonstrating the efficacy of the stable 3D-DCA method in enhancing defect detection accuracy.

The CNR index was calculated for each method. Table 5 compares the maximum CNR values achieved by the different methods, indicating that the proposed stable 3D-DCA method outperformed the other methods in detecting each type of defect, showcasing its exceptional performance in defect detection.

## 5. Conclusions

This paper presents a stable 3D-DCA method, which is a novel approach for analyzing the ultrasonic testing data of composite materials. This method differentiates itself from traditional techniques such as PCA and DAE by accurately capturing the 3D spatiotemporal features embedded in ultrasonic data while simultaneously reducing noise levels. An advantage of a stable 3D-DCA is its improved ability to delineate defect boundaries with enhanced precision, which is a critical factor in ultrasonic data analysis. The shape, location, and depth of the defects were accurately predicted by designing a receptive field to perceive the feature information of the data. Application to a CFRP specimen demonstrates that stable 3D-DCA effectively detects defects, illustrating the feasibility of the method. This method enables earlier defect detection and maintenance, thereby extending the service life of the composite materials. We anticipate that this approach will facilitate the development of various 3D-AE methods for the ultrasonic detection of industrial defects. Further exploration will focus on calculating the depth of the defects using a receptive field with 3D convolutional networks.

## Figures and Tables

**Table 1:** RF calculations.

Layers	Input	Kernel	Stride	Output	RF
Conv1	500 × 57 × 241	3 × 3 × 3	1 × 1 × 1	500 × 57 × 241	3 × 3 × 3
AvgP1	500 × 57 × 241	3 × 3 × 3	2 × 1 × 1	250 × 57 × 241	5 × 5 × 5
Conv2	250 × 57 × 241	3 × 3 × 3	1 × 1 × 1	250 × 57 × 241	9 × 7 × 7
AvgP2	250 × 57 × 241	3 × 3 × 3	2 × 1 × 1	125 × 57 × 241	13 × 9 × 9
Conv3	125 × 57 × 241	3 × 3 × 3	1 × 1 × 1	125 × 57 × 241	21 × 11 × 11
AvgP3	125 × 57 × 241	3 × 3 × 3	2 × 1 × 1	64 × 57 × 241	29 × 13 × 13
Conv4	64 × 57 × 241	3 × 3 × 3	1 × 1 × 1	64 × 57 × 241	45 × 15 × 15
AvgP4	64 × 57 × 241	3 × 3 × 3	2 × 1 × 1	32 × 57 × 241	53 × 17 × 17
Conv5	32 × 57 × 241	3 × 3 × 3	1 × 1 × 1	32 × 57 × 241	69 × 19 × 19
AvgP5	32 × 57 × 241	3 × 3 × 3	2 × 1 × 1	16 × 57 × 241	85 × 21 × 21
Conv6	16 × 57 × 241	3 × 3 × 3	1 × 1 × 1	16 × 57 × 241	117 × 23 × 23
AvgP6	16 × 57 × 241	3 × 3 × 3	2 × 1 × 1	8 × 57 × 241	149 × 25 × 25
MaxP7	8 × 57 × 241	3 × 3 × 3	1 × 1 × 1	8 × 57 × 241	213 × 27 × 27

**Table 2:** Information on defects.

Specimen	Defect	Shape	Area (mm^2^)	Layer
CFRP defect specimen	h1	Square	400	50
	h2		400	30
	h3		400	10
	h4		400	60
	h5		400	40
	h6		400	20

**Table 3:** Defect depth order.

	h1	h2	h3	h4	h5	h6
True depth order	5	3	1	6	4	2
Predicted depth order	5	3	1	6	4	2

**Table 4:** IoU of defects at different locations.

Method	h1	h2	h3	h4	h5	h6	Mean
PCA	0.549	0.663	0.901	0.163	0.802	0.350	0.571
DAE	0.828	0.808	0.675	0.796	0.063	0.315	0.581
3D-DCA	0.790	0.865	0.737	0.712	0.738	0.764	0.768
Stable 3D-DCA	0.839	0.803	0.804	0.684	0.826	0.846	0.800

**Table 5:** CNR of defects at different locations.

Method	h1	h2	h3	h4	h5	h6	Mean
PCA	2.188	2.098	2.203	1.812	1.963	2.151	2.069
DAE	5.136	4.472	4.886	3.540	0.001	4.721	3.793
3D-DCA	5.198	4.304	4.818	3.605	3.938	4.636	4.417
Stable 3D-DCA	7.705	6.778	6.972	5.347	5.728	6.749	6.547

## Data Availability

The data presented in this study are available on request from the corresponding author. The data are not publicly available due to privacy.
